# Knowledge, Attitude, and Practice Regarding the Risk of Diabetic Feet Among Diabetic Patients in the Madinah Region, Saudi Arabia

**DOI:** 10.7759/cureus.49933

**Published:** 2023-12-04

**Authors:** Ahmed S Metwally, Ziyad A Aljohani, Mohammed I Maashi, Abdulaziz A Alrehaili, Basel M Alhejaili, Ahmed M Aljabri, Mohammed A Sindi, Moayed H Alahmadi

**Affiliations:** 1 Department of Family and Community Medicine, College of Medicine, Taibah University, Medina, SAU; 2 Department of Family Medicine, Faculty of Medicine, Suez Canal University, Ismailia, EGY; 3 College of Medicine, Taibah University, Medina, SAU

**Keywords:** practice, attitude, knowledge, diabetic foot, diabetes milletus

## Abstract

Introduction

A patient suffering from diabetes mellitus (DM) has a high chance of developing a diabetic foot. Awareness and attitude toward the prevention of diabetic foot ulcers are necessary for a better quality of life. Educating patients with diabetes about the methods of foot care plays an important role in preventing diabetic foot complications in individuals with DM. This study aims to determine the level of knowledge, attitude, and practices of diabetic patients about the risk of diabetic feet in Madinah, Saudi Arabia.

Methodology

This is an observational cross-sectional study conducted using an online questionnaire. Participants were older than 18 years of age. IBM SPSS Statistics for Windows, Version 27 (Released 2020; IBM Corp., Armonk, New York, United States) was used for data analysis. The questionnaire included patient personal data, patients’ knowledge regarding diabetic feet, attitude, and practice regarding the risk of diabetic feet among diabetic patients.

Results

A total of 1155 participants completed the questionnaire. Exactly 79.9% of people had good knowledge regarding diabetic feet and their care. The analysis revealed that age, gender, education level, and family history of DM are all significant predictors of knowledge levels among the participants. The majority of participants strongly agreed that diabetic patients should promptly seek medical assistance in case of any foot infections (90.8%) and take regular checkups (76.5%). Regarding foot care practices, people generally take good care of their feet but do not seek checkups until they experience symptoms (81.8%).

Conclusion

The study showed that participants had good knowledge about diabetic feet and a careful attitude toward them. Their practices toward diabetic feet were acceptable; however, the issue of late medical consultation after the development of symptomatology needs to be addressed.

## Introduction

Diabetes mellitus (DM) is a long-term metabolic disease marked by high blood glucose, which over time can seriously harm the heart, blood vessels, eyes, kidneys, and nerves [1]. The anticipated prevalence of diabetes worldwide in 2019 is 9.3% (463 million people), and it is expected to increase to 10.2% (578 million) by 2030 and 10.9% (700 million) by 2045. As a result, it is predicted that by 2030, there will be 54% more diabetic patients and a 38% higher mortality rate. In Saudi Arabia, over 25% of the adult population suffers, and by 2030, that number is expected to be more than double [2]. Patients with DM are more susceptible to different complications, including heart disease, hypertension, stroke, kidney disease, loss of vision, nervous system disease, dental disease amputations, and gestational complications. Knowledge and awareness of DM and the related risk factors, complications, and management are necessary for better DM control and a higher quality of life. Unfortunately, many DM patients don't recognize they have the illness until they have a serious complication [3]. One of the effects of diabetes is a diabetic foot, which is caused by peripheral artery disease (PAD) and sensory neuropathy in the feet of diabetic patients [4,5]. The prevalence of diabetic foot ulcers is currently reported to be 6.3% worldwide [6]. It is greater in men (4.5%) and type 2 diabetics (6.4%) and is highest in North America when compared to other regions [6]. However, Saudi Arabia's prevalence of diabetic foot illness is similar to that of other countries [7]. More than two-thirds of non-traumatic lower limb amputations are estimated to be preceded (84%) by ulcers, a crucial event that creates a window for early management [8]. The patients with diabetic foot ulcers were older, had a lower BMI, had been diabetic for a longer period of time, and had higher levels of hypertension, diabetic retinopathy, and a history of smoking than the patients without diabetic foot ulcers [9]. One of the simplest, least expensive, and most efficient ways to prevent foot issues in diabetics is to carefully examine their feet on a regular basis. The most prevalent risk factors for limb loss must be understood in order to properly care for the diabetic foot. A thorough but brief examination of the foot and detailed details of the history can help identify several of these risk factors [10]. Currently, there are not a lot of studies evaluating the degree to which DM patients in Madinah, Saudi Arabia, are aware of diabetic foot. Considering the unsettling increase in DM cases in Saudi Arabia, this study was carried out to determine the knowledge, attitude, and practices (KAP) of diabetic patients about diabetic feet in Madinah, Saudi Arabia.

## Materials and methods

Study design

This is an observational analytic (cross-sectional) study.

Study setting and duration

The study was conducted from the 25th of May 2023 to the 25th of October 2023 in the Madinah region, Saudi Arabia using a predesigned validated questionnaire obtained from a study conducted in the region of Aseer, Saudi Arabia [11].

Study population and sampling

Inclusion Criteria

Diabetic patients who live in the region of Madinah, Saudi Arabia were included in the study.

Exclusion Criteria

Patients who had amputated feet, diabetic feet, foot ulcers, or who did not complete the study questionnaire were excluded from the study.

Sampling Size

The sample size was estimated using the OPENEPI website. The anticipated frequency was 67.4. It was obtained from a previous study conducted in the region of Aseer, Saudi Arabia [11]. The confidence level is 95%, the margin of error is 5%, and the sample size is 338. This sample size will be increased to more than 700 to overcome the incomplete surveys.

Sampling Method

The relevant data was collected using an electronic-designed data-collecting questionnaire in Arabic and English. The confidentiality and privacy of the collected data and participants were kept anonymous for medical and ethical purposes.

Measurements

For measurement tools, we used a predesigned, validated questionnaire for the same research purpose. The questionnaire involves 27 questions. The questionnaire included four sections: patient personal data, patients' knowledge, patients' attitudes, and patients' practice.

Ethical considerations

This study was submitted to Taibah University, College of Medicine Research Ethics Committee (CM-REC), for ethical review, and approval was obtained on 20/05/2023 with this study ID (TU-026-22). The Declaration of Helsinki was followed in the compilation of the study. No personally identifiable information was gathered. The participants were informed of the purpose of the study. They were free to choose to participate in the study or not. The participants were made aware that the information they provided would be kept private and utilized exclusively for the study. We didn't provide the participants with any gifts or incentives.

Statistical analysis plan

We collected, analyzed, and coded the data by using IBM SPSS Statistics for Windows, Version 27 (Released 2020; IBM Corp., Armonk, New York, United States). Numerical variables were described in terms of mean and standard deviation, and for categorical variables, we used numbers and percentages. A p-value less than 0.05 is considered statistically significant. A thorough statistical analysis was carried out on the dataset, incorporating both descriptive and inferential methods. We examined the sociodemographic characteristics of the participants and practice variables by calculating simple frequencies and percentages, and we systematically presented the results in tables. Regarding the knowledge of participants about diabetic feet, we assigned a value of '1' for "yes" as a correct answer and '0' for "no" and "don’t know" as incorrect answers. We then summed up the responses to all questions, categorizing participants with more than 50% correct answers as having a high level of knowledge and vice versa. Similarly, for attitude, we assigned a value of '1' for "agree" for the first three questions and "disagree" for the last two questions as correct answers, while assigning '0' for corresponding responses to all questions as wrong answers. Again, we summed up the responses, categorizing participants with more than 50% correct answers as having a positive attitude, and vice versa. The association between sociodemographic variables and knowledge and attitude scores about diabetic foot was assessed using the Chi-Square or Fisher’s exact test. For further analysis, we employed binary logistic regression to identify adjusted predictors for high vs. low knowledge and positive vs. negative attitude. Statistical significance is set at a p-value of 0.05 or lower, and we calculated a 95% confidence interval.

## Results

The study involved 1155 participants from the general population of Saudi Arabia. Slightly more than half of the participants were female (54.5%), aged between 18 and 40 years (53.2%), and married (52%). Most of them live in Madinah (84.9%), with a substantial proportion holding at least a university degree (70.8%). Approximately 74.2% of the participants had a family history of diabetes mellitus, while nearly 70.9% revealed that they were not clinically diagnosed with diabetes. Among those individuals who had been diagnosed with diabetes (29.1%), only 7.4% reported signs of diabetic foot complications (Table 1).

**Table 1:** Descriptive Statistics of Sociodemographic Variables (n= 1155) n: Number, %: Percentage

Variable		n (%)
Age (years)	< 18	57 (4.9)
	18-40	614 (53.2)
	41-50	211 (18.3)
	>50	273 (23.6)
Gender	Male	525 (45.5)
	Female	630 (54.5)
City of residence	Madinah	981 (84.9)
	Other	174 (15.1)
Height (cm)	< 160	370 (32.0)
	160-170	457 (39.6)
	170-180	268 (23.2)
	>180	60 (5.2)
Weight (kg)	< 60	342 (29.6)
	61-80	493 (42.7)
	81-100	253 (21.9)
	>100	67 (5.8)
Marital status	Single	554 (48.0)
	Married	601 (52.0)
Education level	Illiterate	10 (0.9)
	1ry school	18 (1.6)
	Middle School	44 (3.8)
	High school	265 (22.9)
	Graduate	818 (70.8)
Family history of DM	Yes	857 (74.2)
	No	298 (25.8)
Diagnosis with DM	Yes	336 (29.1)
	No	819 (70.9)
Diabetic foot	Yes	85 (7.4)
	No	1070 (92.6)

**Figure 1:**
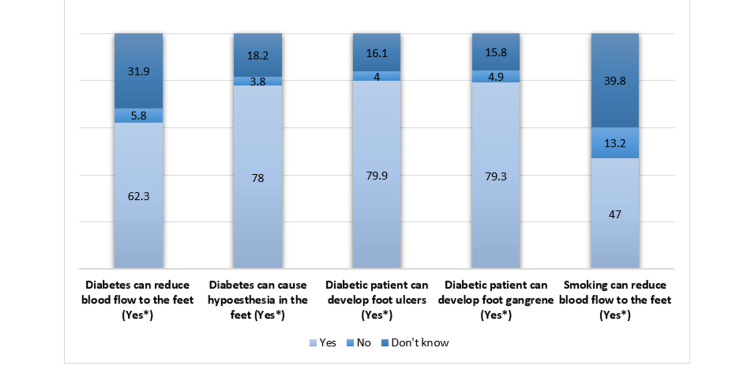
Knowledge About the Diabetic Foot *: Correct answers

To evaluate the participants' knowledge of diabetic feet, we presented them with five relevant questions. The overall responses indicate a high level of knowledge among the participants. They correctly agreed that diabetic patients can develop foot ulcers (79.9%) and gangrene (79.3%). Moreover, they recognized that diabetes could lead to reduced sensation (78%) and blood flow (62.3%) in the lower extremities. However, a relatively smaller percentage were aware that smoking acts as a confounding risk factor for decreased blood flow to the feet (Figure 1).

**Figure 2:**
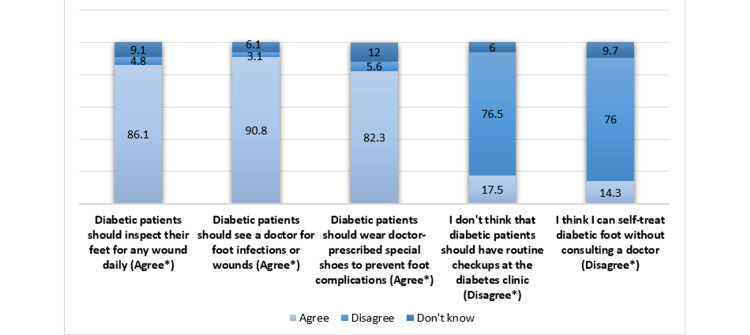
Attitudes Toward the Diabetic Foot *: Correct answers

We also explored the participants' attitudes toward this critical medical condition, both positive and negative. The majority of participants strongly agreed that diabetic patients should promptly seek medical assistance in case of any foot infections or wounds (90.8%), regularly examine their feet for potential wounds (86.1%), and wear special shoes as prescribed by their doctors to prevent diabetic foot complications (82.3%). Similar proportions of participants strongly disagreed with the notion that regular checkups for diabetic patients were unnecessary (76.5%) and rejected the idea of self-managing diabetic foot issues without consulting a doctor (76%) (Figure 2).

Regarding the reported practices concerning diabetic foot care, the results revealed interesting insights into participants' behaviors. A large majority (93.5%) reported washing their feet daily, with 45.3% sometimes and 38% always wearing cotton socks. Interestingly, nearly half (49.3%) sometimes walk barefoot, and 21.7% usually do so. A significant portion (37.7%) did not change their slippers until they were damaged, while the rest of them replaced them once (18.4%) or more (44%) per year. Most participants (81.8%) do not seek foot checkups until they experience symptoms of a disease, and almost 65.8% of patients with DM consult a medical doctor upon finding abnormalities on their feet (Table 2).

**Table 2:** Diabetic Patients Practices Regarding Diabetic Foot Care (n= 1155) n:  Number, %: percentage

Question:		n (%)
Do you wash your feet daily?	Yes	1080 (93.5)
	No	75 (6.5)
Do you continuously wear cotton socks?	Yes	439 (38.0)
	Sometimes	523 (45.3)
	No	193 (38.0)
Do you walk barefoot frequently?	Yes	251 (21.7)
	Sometimes	569 (49.3)
	No	335 (29.0)
How often do you change your footwear?	When slippers are damaged	435 (37.7)
	Once/year	212 (18.4)
	More than once/year	508 (44.0)
How often do you go for foot checkups?	Once/month	51 (4.4)
	Once/6 month	111 (9.6)
	Once/ year	48 (4.2)
	Only when I am ill	945 (81.8)
What would you do if you find an abnormality on your feet?	Manage it myself	395 (34.2)
	Consult a doctor	760 (65.8)

We examined the potential associations between sociodemographic variables and the participants' knowledge levels using the Chi-square/Fisher test. The results indicated statistically significant differences among various age groups in terms of their knowledge scores (p-value<0.001). Notably, more than half of the highly knowledgeable individuals (50.9%) fell within the age range of 18-40 years, while those under 18 years old displayed the lowest awareness of diabetic foot (3%). In terms of gender, there was a statistically significant difference in knowledge scores between men and women (p-value = 0.013). Women (56.6%) demonstrated greater knowledge than men (43.4%) regarding diabetic feet. Marital status also played a role in determining knowledge scores (p-value < 0.001). Married individuals (55.3%) achieved higher knowledge scores compared to single individuals (44.7%). Education levels also had a significant impact on knowledge levels (p-value < 0.001). Graduate participants represented the majority with the highest knowledge scores (73.5%) regarding diabetic foot, while the illiterate participants had the lowest knowledge levels (1%). Moreover, there was a significant difference in the knowledge level based on whether participants had a positive family history of diabetes mellitus (p-value < 0.001). Individuals with a positive family history of DM had a higher knowledge level (78.3%) than those without such a history (21.7%). However, the variables of being diagnosed with DM and the presence of a diabetic foot did not show statistically significant differences between their respective categories in terms of knowledge scores (p-value > 0.05) (Table 3).

**Table 3:** Knowledge Levels Among Participants, Grouped by Sociodemographic Factors (n=1155) *: Chi-square/Fisher test, p< 0.05 is significant

Variable		Low Knowledge (n= 279)	High Knowledge (n= 876)	p-value^* ^
		n (%)	n (%)	
Age (years)	< 18	31 (11.1)	26 (3.0)	<0.001
	18-40	168 (60.2)	446 (50.9)	
	41-50	43 (51.4)	168 (19.2)	
	>50	37 (13.3)	236 (26.9)	
Gender	Male	145 (52.0)	380 (43.4)	0.013
	Female	134 (48.0)	496 (56.6)	
Marital status	Single	162 (58.1)	392 (44.7)	<0.001
	Married	117 (41.9)	484 (55.3)	
Education level	Illiterate	4 (1.4)	6 (0.7)	<0.001
	1ry school	9 (3.2)	9 (1.0)	
	Middle School	19 (6.8)	25 (2.9)	
	High school	73 (26.2)	192 (21.9)	
	Graduate	174 (62.4)	644 (73.5)	
Family history of DM	Yes	171 (61.3)	686 (78.3)	<0.001
	No	108 (38.7)	190 (21.7)	
Diagnosis with DM	Yes	79 (28.3)	257 (29.3)	0.763
	No	200 (71.7)	619 (70.7)	
Diabetic foot	Yes	23 (8.2)	62 (7.1)	0.599
	No	256 (91.8)	814 (92.9)	

Predictors of knowledge levels were further examined through logistic regression analysis. The analysis revealed that age, gender, education level, and family history of DM are all significant predictors of knowledge levels among the participants (p-value < 0.05). These factors had a notable impact on participants' knowledge about diabetic feet. However, marital status, coexisting DM, and having a diabetic foot did not have any influence on predicting knowledge levels related to diabetic feet among the participants. These variables did not emerge as significant predictors in the analysis (Table 4).

**Table 4:** Adjusted Predictors of Knowledge Levels Among Participants *: Binary logistic regression test, p< 0.05 is significant OR: Odds ratio, CI: confidence interval, Hx: history, Dx: diagnosis

	B	p-value^*^	OR (95% C.I)	
				Age (Ref: < 18-40)	-	<0.001	-	
Age (< 18)	-1.104	<0.001	0.332 (0.20-0.45)	
Age (< 41-50)	-0.741	0.005	0.477 (0.28-0.79)	
Age (>50)	-1.753	<0.001	0.173 (0.08-0.36)	
Gender (Ref: Female)	-	-	-	
Male	0.390	0.009	1.477 (1.10-1.97)	
Marital status (Ref: Single)		-		
Married	-0.019	0.915	0.981 (0.68-1.39)	
Education (Ref: Primary school)	-	0.002	-	
Illiterate	-0.298	0.629	0.742 (0.22-2.48)	
Middle School	-1.006	0.188	0.366 (0.08-1.63)	
High School	0.750	0.045	2.116 (1.01-4.39)	
Graduate	0.923	0.012	2.518 (1.22-5.15)	
Family Hx (Ref: No)	-	-	-	
Yes	-0.678	<0.001	0.508 (0.37-0.68)	
Dx with DM (Ref: No)	-	-	-	
Yes	0.116	0.538	1.123 (0.77-1.62)	
Diabetic Foot (Ref: No)	-	-	-	
Yes	0.159	0.585	1.172 (0.66-2.07)	

Similarly, we examined the potential associations between sociodemographic variables and the participants' attitude patterns using the Chi-square/Fisher test. The results indicated statistically significant differences among various age groups in terms of their attitudes toward diabetic foot topics (p-value < 0.001). Notably, more than half of the individuals with a positive attitude (52%) were 18-40 years old, while those under 18 years old displayed the lowest awareness of diabetic foot (4.1%). In terms of gender, there was a statistically significant difference in attitude patterns between men and women (p-value = 0.002). Women demonstrated a positive attitude (56.1%) more than men (43.9%) towards diabetic feet. Marital status also played a role in determining knowledge scores (p-value<0.001). Married individuals (54.1%) showed a positive attitude towards diabetic foot issues compared to single individuals (45.9%). Education levels also had a significant impact on the way of thinking about diabetic feet (p-value<0.003). Graduate participants represented the majority with a positive attitude (72%) regarding diabetic foot, while illiterate participants had the lowest knowledge levels (1.2%). Moreover, there was a significant difference in the perspective of participants toward diabetic feet based on whether participants had a positive family history of diabetes mellitus (p-value = 0.027). Individuals with a positive family history of DM had greater positive inclinations towards diabetic feet (75.2%) than those without such a history (21.7%). Additionally, the presence of diabetic foot showed statistically significant differences with its absence in terms of attitude (p-value < 0.001). The absence of diabetic foot was more associated with a positive attitude towards diabetic foot (94%), compared with having it (6%). However, the variables of being diagnosed with DM showed no significant association with attitude patterns toward diabetic feet (p-value > 0.05) (Table 5).

**Table 5:** Attitude Patterns Among Participants, Grouped by Sociodemographic Factors (n=1155) *: Chi-square/fisher test, p< 0.05 is significant

Variable		Negative Attitude (n= 119)	Positive Attitude (n= 1036)	p-value^*^
		n (%)	n (%)	
Age (years)	< 18	15 (12.6)	42 (4.1)	<0.001
	18-40	75 (63.0)	539 (52.0)	
	41-50	18 (15.2)	193 (18.6)	
	>50	11 (9.2)	262 (25.3)	
Gender	Male	70 (58.8)	455 (43.9)	0.002
	Female	49 (41.2)	581 (56.1)	
Marital status	Single	78 (65.5)	476 (45.9)	<0.001
	Married	41 (34.5)	560 (54.1)	
Education level	Illiterate	2 (1.7)	8 (0.8)	0.003
	1ry school	6 (5.0)	12 (1.2)	
	Middle School	8 (6.7)	36 (3.5)	
	High school	31 (26.1)	234 (22.5)	
	Graduate	72 (60.5)	746 (72.0)	
Family history of DM	Yes	78 (65.5)	779 (75.2)	0.027
	No	41 (34.5)	257 (24.8)	
Diagnosis with DM	Yes	39 (32.8)	297 (28.7)	0.394
	No	80 (67.2)	739 (71.3)	
Diabetic foot	Yes	23 (19.3)	62 (6.0)	<0.001
	No	96 (80.7)	974 (94.0)	

Predictors of attitude patterns were further examined through logistic regression analysis. The analysis revealed that age, gender, education level, and presence of diabetic feet are all significant predictors of attitude patterns among the participants (p-value < 0.05). These factors had a notable impact on participants' attitudes toward diabetic feet. However, marital status, family history of DM, and clinical diagnosis of DM did not have any influence on predicting knowledge levels related to diabetic feet among the participants. These variables did not emerge as significant predictors in the analysis (Table 6).

**Table 6:** Adjusted Predictors of Attitude Patterns Among Participants *: Binary logistic regression test, p< 0.05 is significant OR: Odds ratio, CI: confidence interval, Hx: history, Dx: diagnosis

	B	p-value^*^	OR (95% C.I)	
				Age (Ref: < 18-40)	-	<0.001	-	
Age (< 18)	-1.576	<0.001	0.207 (0.09-0.47)	
Age (< 41-50)	-1.132	0.011	0.322 (0.13-0.76)	
Age (>50)	-2.098	<0.001	0.123 (0.04-0.36)	
Gender (Ref: Female)	-	-	-	
Male	0.578	0.006	1.783 (1.18-2.69)	
Marital status (Ref: Single)		-		
Married	-0.377	0.141	0.686 (0.41-1.13)	
Education (Ref: Primary school)	-	0.011	-	
Illiterate	-0.816	0.248	0.442 (0.11-1.76)	
Middle School	-1.993	0.047	0.136 (0.01-0.97)	
High School	0.521	0.291	1.68 (0.64-4.42)	
Graduate	0.583	0.237	1.791 (0.68-4.70)	
Family Hx (Ref: No)	-	-	-	
Yes	-0.261	0.240	0.770 (0.49-1.19)	
Dx with DM (Ref: No)	-	-	-	
Yes	0.036	0.895	1.037 (0.60-1.76)	
Diabetic Foot (Ref: No)	-	-	-	
Yes	1.518	<0.001	4.565 (2.39-8.70)	

## Discussion

This study aimed to highlight the knowledge of diabetic feet among diabetic patients in Madinah, Saudi Arabia. Also, to identify their attitude toward diabetic foot care and their daily life practices regarding their foot care. DM is prevalent in Saudi Arabia, with over 25% of the adult population suffering from it [2]. Our study, with a sample size of 1155 participants, showed that 29.1% of the population was suffering from DM in Madinah. The prevalence of diabetic foot ulcers is 6.3% worldwide and similar to Saudi Arabia, which makes up about half of the diagnosed diabetic patients [7,12].

According to our study, the prevalence of diabetic feet is greater among the diagnosed diabetics, making it 7.4%. Lower limb amputation is the ultimate treatment for diabetic feet, and 49.6% of the amputations are due to it [13]. About 80% of diabetic feet have to undergo lower limb amputation [14]. We focused on assessing the level of knowledge among people regarding diabetic feet with five questions. The results were surprisingly good, showing that 79.9% had good knowledge about diabetic ulcer complications. The results are higher in comparison to other studies which were 53.6%, 53.3%, 13.2%, and 58% respectively according to our citations [15-18]. However, the sample size of the three aforementioned studies was smaller. Moreover, an almost similar good knowledge result regarding diabetic foot was shown in the study conducted by Alshammari (2019) (76.6%) in Riyadh and by Algshanen et al. (2017), with 70 to 80% good knowledge results [6,19].

In agreement with other studies conducted, our study showed that patients who had a higher level of education had a better knowledge of diabetes foot care (73.5%) [15,18,20]. Illiterate people have the lowest level of knowledge (0.7%). The reason could be the self-administered questionnaire instead of being asked by an interview, as they might require deeper explanations of questions. Married status had an impact on the knowledge score (55.3%), similar to the study conducted in Riyadh [17]. Being married adds spousal support and responsibility for taking care of nutrition, dietary control, management, and treatment compliance for chronic diseases. That’s why married people know more about diabetic feet than unmarried people. Our study signified that people between the ages of 18 and 40 had a good awareness of diabetic foot and its consequences and care (50.9%). Young people were aware that the reduced blood supply and neuropathy could lead to diabetic feet, especially educated young people and those with a family history of diabetes. Our study is consistent with a study by Al Amri et al. (2021) in the region of Aseer [11]. The majority of participants strongly agreed that diabetic patients should promptly seek medical assistance in case of any foot infections or wounds (90.8%), regularly examine their feet for potential wounds (86.1%), and wear special shoes as prescribed by their doctors to prevent diabetic foot complications (82.3%), following the study conducted in the region of Aseer by Al Amri et al. (2021) [11]. Similar proportions of participants strongly disagreed with the notion that regular checkups for diabetic patients were unnecessary (76.5%) and rejected the idea of self-managing diabetic foot issues without consulting a doctor (76%). The current study highlighted that people with a positive family history of diabetes or who have been diagnosed with DM have no statistically significant inclination in their attitude pattern toward diabetic foot complications.

These results are in contrast to the study regarding KAP toward the diabetic foot in Riyadh, representing the lack of active education among the people in Madinah and ignorance in their attitude towards this critical care medical condition [6]. For this reason, physicians, and nurses, regardless of the duration of diabetes, should realize the impact of education and enhancing awareness among visitors to healthcare centers about diabetic complications, such as diabetic foot, at the time of diagnosis [21]. Based on the level of education, gender, and young age, people had positive attitudes toward diabetic foot care. Our study also highlighted that the absence of the diabetic foot was associated with a positive attitude toward the diabetic foot (96%) instead of having it. Awareness of the susceptibility of diabetic feet and active measures to prevent it are separate entities. A possible explanation is that diabetic feet develop slowly over time, which leads them to not realize the gravity of complications [22]. As for practices, 93.5% wash their feet daily, consistent with other studies, with over 80% doing so [11]. About half of them wear cotton socks and walk barefoot, which could aggravate the diabetic foot condition and increase the odds of untreatable wounds and/or infections. Our participants reported that 81.8% do not seek foot checkups until they experience symptoms of the disease. This attitude is similar to the attitude of participants in the Al Amri et al. (2021) study, which is an unhealthy practice [11]. Only 65.8% of people in Madinah reported that they would consult a doctor if they found a foot abnormality, almost similar to the people of the Aseer Region (68.6%) [11]. Timely care and follow-up of diabetic feet with your doctor can prevent many further disease complications at an early level. Involving multi-disciplinary care at an early level seems beneficial and cost-effective in comparison to complicated diabetic foot cases [23].

Limitations

The self-assessment online data collection method could limit the study as compared to a face-to-face interview method in terms of failure to understand the questions. The fact that our questions were of a closed-ended style, in a yes-or-no pattern, might affect the validity of the information. This could also be the reason for the high level of knowledge among our participants. A constraint in this study is its exclusive focus on the residents of Madinah, which restricts the generalizability of findings to a broader sample size or the entire population of Saudi Arabia as the cultures are very different from one area to another.

## Conclusions

In conclusion, the current study showed that 79.9% of participants were knowledgeable about diabetic foot care. High knowledge was found among young people, educated members, married people, and people with a family history of DM. Also, participants had a good attitude toward diabetic feet and their care. Practice regarding diabetic feet was satisfactorily average. However, people should choose to consult their doctors more often. Foot checkup times were not high among the patients. Diabetic patients need periodic health education programs and training for all diabetes-related complications, including diabetic foot-related consequences. These findings can inform targeted educational interventions to enhance awareness and promote better diabetic foot care practices, ultimately reducing the burden of diabetic foot complications in that region.
